# Commonalities between Disaster and Climate Change Risks for Health: A Theoretical Framework

**DOI:** 10.3390/ijerph15030538

**Published:** 2018-03-16

**Authors:** Nicola Banwell, Shannon Rutherford, Brendan Mackey, Roger Street, Cordia Chu

**Affiliations:** 1 Centre for Environment and Population Health, School of Environment, Griffith University, Brisbane 4111, Australia; s.rutherford@griffith.edu.au; 2 Griffith Climate Change Response Program, Griffith University, Gold Coast City 4222, Australia; b.mackey@griffith.edu.au; 3 UK Climate Impacts Programme, Environmental Change Institute, University of Oxford, Oxford OX1 3QY, UK; roger.street@ukcip.org.uk; 4 Centre for Environment and Population Health, School of Medicine, Griffith University, Brisbane 4111, Australia; c.chu@griffith.edu.au

**Keywords:** health, public health, disaster, climate change, disaster risk reduction, climate change adaptation, health impacts, risk, emergency

## Abstract

Disasters and climate change have significant implications for human health worldwide. Both climate change and the climate-sensitive hazards that result in disasters, are discussed in terms of direct and indirect impacts on health. A growing body of literature has argued for the need to link disaster risk reduction and climate change adaptation. However, there is limited articulation of the commonalities between these health impacts. Understanding the shared risk pathways is an important starting point for developing joint strategies for adapting to, and reducing, health risks. Therefore, this article discusses the common aspects of direct and indirect health risks of climate change and climate-sensitive disasters. Based on this discussion a theoretical framework is presented for understanding these commonalities. As such, this article hopes to extend the current health impact frameworks and provide a platform for further research exploring opportunities for linked adaptation and risk reduction strategies.

## 1. Introduction

The health impacts of climate change and climate-sensitive disasters (caused by various climate-sensitive hydrological, climatological, biological and meteorological hazards) are of increasing importance globally [1,2,3,4,5,6,7]. The number of people affected by disasters in 2016 was the highest it has been in the last decade, of which hydrological, meteorological and climatological disasters comprised 90.9% [8]. This does not include those biological hazards which are also influenced by climate change. While the 8733 reported disaster deaths in 2016 was lower than in previous years [8], these mortality rates reflect the tip of the iceberg in terms of health impacts of climate change and disasters [9,10].

Disasters increase mortality and morbidity rates, including health impacts such as injury, toxic exposure, disease, and mental health, among others [5,11,12,13]. These impacts are well documented, as are the potential pathways and endpoints for climate change impacts on health. Climate change may trigger increased morbidity and mortality, as a result of climate-sensitive diseases, extreme weather events, increasing non-communicable diseases, and exacerbating existing health problems [14]. The complex interactions of both climate change and disasters with social, environmental and biological factors on individual and systems levels make their health impacts difficult to measure [9,10,15,16]. The true extent of health impacts of disasters is often underrepresented due to the reliance on mortality rates as indicators of health impacts, complexity of interactions leading to health impacts, and challenges in collecting data during, and in the aftermath of, disaster events [9,10,17,18]. Similarly, health impacts of climate change are notably difficult to measure due to the time and spatial scales over which health impacts arise, the complex pathways that involve both ecosystems and human systems, numerous stakeholder groups involved in coordinating and collecting data, as well as limited baseline data [15,16,19,20]. Not only are there commonalities in the current and potential impacts on health, there are overlaps in the different pathways through which these impacts occur. 

Health impacts of the disasters resulting from hazards (defined in Section 2) have commonly been discussed in terms of two categories, direct and indirect impacts on health and health systems [1,18,21,22]. Similarly, the potential health impacts of climate change have been discussed in terms of direct and indirect pathways since the early nineties [6,16,19,23,24,25,26,27]. Although not all disaster-related health impacts are the result of climate-sensitive hazards or influenced by climate change, and not all climate change impacts are related to disasters, the impacts on human health and health systems have “considerable overlap” [13] (p. 455). 

Previous discussions of these health impacts have not identified the commonalities in direct and indirect health impact pathways between climate change and climate-sensitive disasters. Direct health impacts are those unmediated impacts of climate-sensitive hazards on health [5,6,9,11,12,14,18,21,22,26,27,28,29,30,31], while indirect health impacts are mediated through ecosystems and human systems [5,6,11,14,18,21,28,29,30]. The involvement of health in disaster risk reduction, climate change adaptation, and sustainable development continues to grow internationally [1,2,3,4,32,33,34,35,36]. It is therefore of increasing importance to understand the commonalities in health impact pathways of climate-sensitive disasters and climate change as a starting point for developing joint strategies to adapt to, and reduce, health risks, particularly in resource constrained and highly vulnerable settings.

Therefore, this article discusses commonalities between health impact pathways of climate change and climate-sensitive disasters. It first offers a clarification of key terminology. It then discusses health impacts of disasters and climate change in terms of direct and indirect pathways. Following this, their commonalities in risks to health are presented in a novel framework. The framework was developed by examining similarities in the elements and relationships presented in existing relevant and commonly cited frameworks. Components of these frameworks were adapted to illustrate the common pathways between health risks of climate change and climate-sensitive disasters. Following the framework, recommendations for future research are provided with the view to driving further work. 

## 2. Defining Common Concepts in Disaster and Climate Change Risks to Health

The key concepts related to disaster and climate change risks to health are defined here to provide a conceptual foundation for the discussion of commonalities in health impact pathways. It is important to clearly understand that climate change is a driver for climate-sensitive health hazards. The impact of these hazards with respect to the exposure and vulnerability of a community can result in climate-sensitive disasters. Hazards classified as climate-sensitive within this article include hydrological (e.g., flood and rainfall-triggered landslide), climatological (e.g., drought and wildfire), biological (e.g., relevant climate-sensitive diseases such as dengue, cholera, malaria, etc.) and meteorological hazards (e.g., temperature extremes, severe storms). It is the interaction of hazard, exposure, vulnerability and capacity which result in climate change and disaster risks to health [26,37,38]. These key concepts are defined within the context of disasters and climate change in Table 1. 

## 3. Direct Health Impact Pathways

Direct impacts on health include death, injury, disease, mental health impacts, and other health issues such as toxic exposure that can be directly linked back to a disaster event [5,9,11,12,18,21,28]. Discussions of direct impacts of climate change on health include morbidity and mortality, primarily resulting from changes in climate and weather extremes such as heat, drought, cold weather and rainfall [5,6,14,22,26,27,29,30,31]. Climate and weather extremes are defined here as “the occurrence of a value of a weather or climate variable above (or below) a threshold value near the upper (or lower) ends of the range of observed values of the variable” [26] (p. 116). For example, the frameworks of direct and indirect impacts of climate change and health of both McMichael [16] and Watts et al. [27] suggest that direct impacts of climate change on health originate from climate-related hazards such as storms, droughts, heatwaves and floods. Within the climate change and health literature, it is common that climate-sensitive disasters are referred to as examples within the direct health impacts pathways. However, morbidity as a result of climate change is not always the result of what can be classified as extreme events. Extra morbidity and mortality can occur as a result of climate-sensitive hazards that are anomalous (not extreme) weather. For example, periods of warm or cold weather that deviate from usual conditions which are not classed as climate extremes may have health implications [44,45,46]. Some climate change and health literature also acknowledges the indirect pathways for health impacts of climate-sensitive disasters [27].

## 4. Indirect Health Impact Pathways

Indirect health impacts of disasters can result from unmet primary healthcare needs after a disaster due to the change in healthcare demands, and reduced capacity of the health system to meet these needs and baseline healthcare needs (for example, prenatal care, chronic disease management, etc.) [5,11,18,21,28]. These impacts are also a result of the loss of “normal living conditions” [21] (such as adequate and appropriate shelter), and degradation of “environmental health safeguards” [47] and health-determining sectors and infrastructure that populations and health systems rely on (such as clean water, sanitation, waste management, electricity, etc.) [18,21,28]. For example, outbreaks of diarrhoeal diseases may occur after a disaster or complex emergency when affected populations are living in overcrowded evacuation centres and the water and sanitation infrastructure and practices are degraded [48]. Conversely, acute respiratory infections may occur in susceptible and malnourished populations living in overcrowded evacuation shelters with poor ventilation [48]. Furthermore, adverse mental health effects may occur as the result of physical and emotional trauma, displacement, life stressors after a disaster, and limited social support [49,50,51]. Social and economic consequences of disasters also indirectly impact health, well-being and development [5,28]. Examples of indirect health impacts include worsening of chronic health conditions due to the limited access to health care services, otherwise avoidable maternal deaths, and declining nutritional status due to food shortage [9,18,21].

Similarly, indirect health impacts of climate change occur as a result of impacts of climate change on secondary factors, which in turn result in health impacts. These indirect impacts occur through two processes mediated by ecosystems and/or human systems [5,6,14,29,30]. Ecosystems mediate health impacts of climate change through alterations in characteristics of vegetation, soil, baseline air and water quality, as well as ecosystems services [14]. Health impacts mediated by ecosystems primarily include changes in disease vector distribution and life cycle, water and food-borne diseases, and air quality and pollution [5,14,29,30,52]. Climate change can alter transmission seasons and geographical distribution of climate-sensitive diseases [53] and this is a key difference between the indirect pathways of health impacts from climate change as compared to disasters. However, the environmental mediation of climate change impacts can often involve climate extremes and climate-sensitive disasters in the process. For example, extended areas of increased temperatures and drought are potentially at risk of outbreaks of waterborne diseases where warmer temperatures cause higher proliferation of pathogens [52,54], and higher concentration of pathogens, increased turbidity and contamination, and decreased pressure at treatment plants result in decrease water quality and availability [52,55]. Health impacts mediated by human systems are those which result from the impacts climate change has on social processes and systems [14], such as access to and quality of health care, population growth, aging, socioeconomic status, and the economy [52,56]. They can include: occupational heat stress; mental health impacts as a result of conflict or livelihood loss triggered by climate change; food insecurity and undernutrition as a result of reduced agricultural production; and restricted access to adequate and necessary public and primary health care [5,14,27].

Climate-sensitive disasters and climate change also impact public and primary health services by impacting functionality through damages to infrastructure and non-structural factors, including equipment and medical supplies, and factors relating to the functioning of vital aspects of the health system, such as losses of medical records, losses or death of general and specialised health personnel, etc. [18,21,57,58]. Secondary impacts of disasters on health systems include the suspension of public health efforts due to the redirection of personnel and financial resources to emergency or disaster response on an ongoing basis [21,59]. For example, increases in vector borne disease may occur over the long term due to interruption of vector control programmes following a disaster [60,61]. 

The impacts on health systems and those that support them are not well documented [13]. However, there is potential for climate change to impact health facilities and systems not only through extremes in weather and climate events, but also through sea level rise in the context of exposed coastal facilities, as well as increasing burden on the health system through climate-sensitive diseases, such as dengue, malaria, or non-communicable diseases. As a result, these impacts of climate change and disasters on health systems can cause additional indirect health impacts. While these areas are not as well documented as the population health impacts [13,57,62,63], health systems and the systems that support health is becoming an area of increasing concern. The impacts of climate change and disasters on health systems can be categorised in terms of:

- Changes in volume and patterns of health service demand from increased morbidity and mortality [15,21,58,64,65];
- Reduced capacity to meet the primary and public health needs due to interruption of vital components of the health system (e.g., infrastructure or equipment damage, reduced staff) [13,15,18,21,58,64]; and
- Interruption of health-determining services such as access to clean water, sanitation, food, energy, communication, etc. [58,64,65].

## 5. Framework for Understanding Commonalities in Health Risks of Climate Change and Climate-Sensitive Disasters

**Figure 1:**
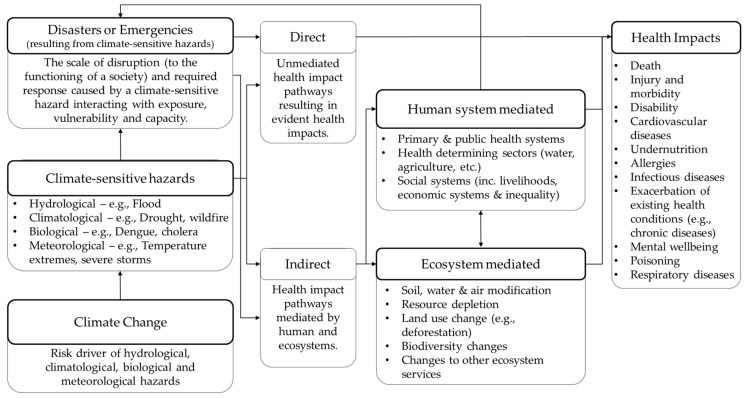
Direct and indirect health impact pathways of climate change and climate-sensitive disasters. Developed based on frameworks contained in [13,16,24,27,67].

The commonalities between health impact pathways of climate change and climate-sensitive disasters can be understood when climate-sensitive disasters are considered as their risk component parts, i.e., where the health of vulnerable communities is impacted by exposure to hazards. Understanding the relationship between climate change and the hydrological, climatological, biological and meteorological hazards that result in climate-sensitive disasters is fundamental for understanding the similarities in the resulting health impacts and the required actions to address them. Figure 1 provides a novel framework for identifying the commonalities in direct and indirect pathways of climate change and climate-sensitive disaster impacts on health, where previously these impact pathways have been considered separately. The commonalities in impact pathways include the influences of climate change as a risk driver for health impacts caused by increasing the frequency and intensity of climate-sensitive hydrological, climatological, biological and meteorological hazards [35,66]. 

In understanding these commonalities, the distinction between disaster and emergency is important. The authors recognize that the term emergency is at times seen as synonymous with the term disaster [37], in the context of this commentary, emergencies are defined as events that “*do not result* in the *serious* disruption of the functioning of a community or society” [37] (p. 13), the key distinction being the level of disruption encountered and if that disruption exceeds current capacity to respond. Climate-sensitive disasters and emergencies are again distinguished from climate-sensitive health hazards which do not evolve into emergencies or disasters, but still impact health. In this framework, climate change has potential impacts on health both as a driver of climate-sensitive disaster and emergency risk, as well as through climate-sensitive hazards that do not evolve into disasters. This distinction demonstrates the commonalities in health impact pathways of climate-sensitive hazards. It also considers both the variation in severity of the disruption and whether coping capacity is exceeded. In doing so, the framework recognises that not all climate-sensitive hazards result in disasters or even emergencies. The authors note, therefore, that the severity of health impacts may differ and thus so must the scale of the response. The list of health impacts and mediating factors provided in the framework are not exhaustive as they are intended as a thematic scaffold to guide understanding.

In this framework hazards (as defined in Table 1) should not be understood in the conventional sense the concept is used in disaster risk management. This framework characterizes hazards as climate-sensitive events that pose risks to human health. This highlights the commonalities in health hazards of concern to practitioners working with climate change and climate-sensitive disaster impacts on health. A distinction is made between hydrological, climatological, biological and meteorological hazards based on the EM-DAT hazard classification [68]. This is to ensure a holistic approach to understanding climate-sensitive health hazards, similar to the all hazards approach now embraced within disaster risk reduction and management. This enables the inclusion of and hazards that have emerged as potentially climate-sensitive, such as weather and climate extremes, and some biological hazards. For example, this broader frame comprises relevant climate-sensitive infectious diseases as hazards, where traditionally these were not considered hazards within disaster risk reduction and management. Furthermore, distinguishing between the different types of health hazards illustrates the extensive range of commonalities in health impact pathways. For example, this classification of hazards ensures that meteorological hazards such as heatwaves are understood as health hazards from both a climate change and disaster perspective, even if the classification of heatwaves as a disaster or emergency occurs in a small portion of experienced heatwaves [69]. It also includes hydrological hazards such as flood and landslides, as well as climatological hazards such as wildfire and drought. In doing so, this allows the framework to also account for climate change-related morbidity and mortality that results from hazards not classified as extreme events or disasters. For example, heat-related health impacts are expected to increase as a result of anomalous changes in temperature which are not classed as extreme events [44,46], such as diurnal temperature variation [45]. Finally, the types of hazards enable the consideration of both acute hazards, such as outbreaks with potential for rapid proliferation and development into a health emergency or disaster, and slow-onset hazards, such as droughts or increasing dengue prevalence. The incorporation of slow-onset hazards broadens the conventional frame of hazards and enables the consideration of those which have conventionally not been considered as potentially leading to disasters. These slow-onset hazards impact health through similar direct and indirect pathways, however this occurs at a different temporal scale to acute hazards.

Finally, this framework incorporates an innovative interpretation of the potential hazard–disaster–impact relationship to allow for the inclusion of escalating health impacts. The framework incorporates a cyclic aspect where climate-sensitive hazards may have impacts on health but these do not necessarily evolve into disasters until after they are mediated by ecosystems or human systems. For example, the initial outbreak of Ebola in 2014 impacted human health but was not seen as an emergency or disaster until after interacting with complex human systems. For example, the limited cultural and religious acceptability of scientific methods for combating transmission worsened the health risk and impact, and thus evolving into health emergencies [70]. While attributing any singular disease outbreak to climate change is not possible, the Ebola outbreak is given as an example of the complex feedback loop which can result in health impacts from climate-sensitive hazards escalating to emergencies and disasters. Here, Ebola is considered to be a potential climate-sensitive hazard as the proliferation of the virus is potentially influenced by climate-sensitive factors such as climatological and meteorological conditions including periods of unusual drought and rain [71,72,73]. This draws attention to the consideration of a sub-group of infectious diseases which are potentially climate-sensitive but have not yet been identified as such due to current spatial and temporal limitations in meteorological and baseline health data [72]. 

In addition to the commonalities identified here between in the indirect and direct health impact pathways, there are of course important differences in these pathways. First, the impacts from climate-sensitive disasters and climate change may occur over different time scales ranging from acute events such as typhoons, to slow onset disasters such as sea level rise and droughts exacerbated by climate change [26]. Second, the spatial scales over which health impacts arise may differ, for example climate change can alter the geographic distribution of climate-sensitive diseases [14], whereas climate-sensitive disasters such as a tropical storm are location specific [13]. Third, not all disasters are climate change related, just as not all climate change impacts on health are the result of disasters. Disasters caused by earthquakes or volcanic eruptions are classified as geophysical hazards and distinguished from those known to be caused by climate-sensitive hazards. However, the authors acknowledge that these hazards also result in direct and indirect health impacts. Likewise, anthropogenic disasters such as terrorist attacks and conflict are distinguished from climate-sensitive hazards. Similarly, not all climate change impacts on health are related to disasters. This includes health impacts from some slow-onset hazards such as health risks from salinization of drinking water resulting from sea level rise not linked to storm surge [74,75]. However, these hazards and their associated direct and indirect health impact pathways are not captured in this framework as this is outside of its intended scope.

## 6. Conclusions

The direct and indirect health impacts of climate change and climate-sensitive disasters are a growing concern for human health [13]. The health impacts pathways of both climate-sensitive disasters and climate change have numerous commonalities [5,6,9,11,12,14,18,21,28,29,30,47,76] suggesting the potential for synergist and complementary actions. However, the commonalities between these pathways have not previously been discussed. Considering climate-sensitive disasters in terms of their risk components (hazard, vulnerability, exposure and adaptive capacity) enables clear identification of the commonalities in the health impact pathways of disasters and climate change, where climate change is understood as a driver of hazard frequency and intensity. The framework presented here identifies climate change as a risk driver for many hydrological, climatological, biological and meteorological hazards. These climate-sensitive hazards have the potential to directly and indirectly impact health. They also have the potential to develop into disasters through interactions with various degrees of a community’s exposure, vulnerability and adaptive capacity. Subsequently, these disasters also directly and indirectly impact health. 

The commonalities in health impact pathways show a clear link between climate-sensitive disaster and climate change risks in health. With this knowledge, it is also reasonable to assume that the strategies in place, or being developed, to reduce these risks must also have numerous commonalities. This could include actions that address environmental and social determinants of health pertinent to climate-sensitive hazards; increase preparedness and response to both climate change and climate-sensitive disaster risks; develop joint climate information services informing health programmes and services; integrate information services, and risk communication and early warning systems; and strengthen health systems resilience. The intent of this framework is to indicate areas of similarity and overlapping interest for health professionals to facilitate discussions around joint adaptation and risk reduction actions for health. By identifying common health risks, this framework takes the first step in developing a common language for identifying and addressing health risks in both the disaster risk reduction and climate change adaptation spaces. The authors acknowledge that data and analysis techniques are not yet sufficient to directly attribute climate change to all climate-sensitive hazards. However, addressing common health risks of both climate change and climate-sensitive disease facilitates a no-regrets approach to adaptation and risk reduction. 

Finally, this article contributes to current literature calling for links between disaster risk reduction and climate change adaptation strategies in health. Since there is a paucity of research examining links between disaster risk reduction and climate change adaptation in health, there is a need to strengthen research to develop a greater understanding of the opportunities and barriers for building these links in not only policy, but also implementation. To this end, by examining commonalities in health risks, this article should be useful in facilitating joint adaptation and risk reduction strategies which safeguard population health.

## Figures and Tables

**Table 1:** Key terms related to disaster and climate change impacts on health.

	Disaster	Climate Change	Common Elements
Term	The interaction of a hazard with exposure, vulnerability and capacity resulting in *serious* disruption, losses and impacts to a community [37].	Changes in climate mean and/or variability that persist over long periods [38].	
Risk	Potential interaction of hazard, exposure, vulnerability and capacity that present the possibility for losses or impacts on a population and elements of a society [37].	The result of the interaction of vulnerability (including capacity), exposure and hazard [26,38].	Interaction of vulnerability (including capacity), hazard and exposure.
Exposure	Elements of communities, infrastructure, organisations or systems that are located within the proximity of a hazard, thus potentially subject to damage and loss [37].	Existence of elements of human and ecosystems in places and settings which could be adversely affected by climate change [38].	Presence of system elements in locations which will be potentially impacted by hazards.
Hazard	An event (geophysical, hydrological, climatological, biological, meteorological, technological or human induced) that has the potential to cause losses to human and ecosystems [37].	Natural or human-induced events that have the potential to occur in the future and impact exposed and vulnerable aspects of a system [26].	Interaction of hazard with exposure and vulnerability.
Vulnerability	“Susceptibility of an individual, a community, assets or systems to the impacts of hazards” [37] (p. 24), caused by economic, social, physical and environmental factors [37,39,40,41,42,43].	Potential to be adversely affected, including factors such as susceptibility, predisposition and capacity [26,38].	Susceptibility to potential adverse effects.
Capacity	Ability of individuals, communities, organisations and systems to access and use the skills and resources to reduce or manage disaster risk [37,39].	Individual, community, societal, or organizational strengths, attributes, and resources that enable responses to change [26,38].	Use of resources and skills to address risks.
